# High stability of electro-transport and magnetism against the A-site cation disorder in SrRuO_3_

**DOI:** 10.1038/srep27840

**Published:** 2016-06-14

**Authors:** Y. L. Wang, M. F. Liu, R. Liu, Y. L. Xie, X. Li, Z. B. Yan, J.-M. Liu

**Affiliations:** 1 Laboratory of Solid State Microstructures and Innovation Center of Advanced Microstructures, Nanjing University, Nanjing 210093, China; 2 Institute for Advanced Materials, Hubei Normal University, Huangshi 435002, China

## Abstract

It is known that the electro-transport and magnetism of perovskite alkaline-earth ruthenate oxides are sensitive to the lattice distortion associated with the A-site cation size. Orthorhombic CaRuO_3_ and cubic BaRuO_3_ exhibit distinctly different electro-transport and magnetic properties from orthorhombic SrRuO_3_. It has been suggested that SrRuO_3_ can be robust against some intrinsic/external perturbations but fragile against some others in terms of electro-transport and magnetism, and it is our motivation to explore such stability against the local site cation disorder. In this work, we prepare a set of SrRuO_3_-based samples with identical averaged A-site size but different A-site cation disorder (size mismatch) by Ca and Ba co-substitution of Sr. It is revealed that the electro-transport and magnetism of SrRuO_3_ demonstrate relatively high stability against this A-site cation disorder, characterized by the relatively invariable electrical and magnetic properties in comparison with those of SrRuO_3_ itself. A simple electro-transport network model is proposed to explain quantitatively the measured behaviors. The present work suggests that SrRuO_3_ as an itinerant electron ferromagnetic metal possesses relatively high robustness against local lattice distortion and cation occupation disorder.

As the knowledge at textbook level, transition metal oxides with typical compact ABO_3_ perovskite structure as a representative class of correlated electron systems promise emergent electronic and magnetic phenomena such as colossal magnetoresistance effect in manganites, high-*T*_*c*_ superconductivity in cuprates and iron-based systems, and multiferroic effects in various transition metal compounds^1^,^2^,^3^,^4^,^5^. Basically, most of these materials belong to the 3*d* transition metal oxides where electron correlation is usually strong, making the electronic structure relatively localized and the associated quantum states controllable technically^6^,^7^. Some more complicated effects and emergent phenomena would occur in the 4*d* transition metal oxides where the electron correlation (*U*) is comparable with the electron bandwidth (*W*)^8^,^9^,^10^,^11^,^12^. Two major features of the 4*d* oxides with respect to 3*d* oxides are the relatively weaker electron correlation and the more extended *d*-orbitals. In these cases, the competition between the correlation and electron kinetic energy is marginal, allowing more fascinating consequences in terms of quantum states and magneto-transport behaviors against intrinsic and extrinsic stimuli, even if these stimuli are weak^8^,^10^. The most studied perovskite ruthenates are ARuO_3_ with A = Sr, Ca, and Ba^13^,^14^,^15^,^16^,^17^, although other ruthenates like A_2_RuO_4_ and A_3_Ru_2_O_7_ *etc* also exhibit rich quantum phase transitions and exotic states including superconductivity^18^,^19^,^20^. We shall come back to the simplest cases of perovskites ARuO_3_ to outline the underlying physics.

For ABO_3_ perovskites, the ionic size mismatch between the A and B sites is the origin for lattice symmetry variation and structural distortions realized by oxygen octahedra rotation and/or tilting, which are often discussed within the framework of structural tolerance factor^21^,^22^. Therefore, the B-O-B bonds may no longer be straight but bent according to some coherent distortion mode such as GdFeO_3_-type mode^16^,^23^. Since the bond angle (ϕ) controls the charge hybridization between the B cation *d*-orbitals and O 2*p*-orbitals, the electronic structure and magnetism can be remarkably dependent of the A-site cation and its radius (*R*_A_). The perovskites ARuO_3_ manifest the physics of electron correlation within this framework too^9^,^16^. Structurally, SrRuO_3_ (SRO) and CaRuO_3_ (CRO) both exhibit the orthorhombic (o-) structure^9^,^14^,^15^,^24^,^25^. However, BaRuO_3_ (BRO) may have poly-type structure depending on the synthesis details^14^,^26^,^27^,^28^,^29^. A synthesis of BRO at ~1000 °C under ambient pressure or a pressure below ~10 GPa may produce the nine-layered rhombohedral (9L), four-layered hexagonal (4 h), or six-layered hexagonal (6 h) structure^27^,^29^. A cubic (c-) BRO perovskite (3C) structure can only be synthesized above ~18 GPa^14^,^30^,^31^.

For a consideration of structural consistence, we confine our discussion on the o-SRO and o-CRO as well as c-BRO in the present work. The lattice structures of these compounds are shown in Fig. 1 for a guide of eyes. The coherent distortions of oxygen octahedra framework can be clearly seen for o-SRO and o-CRO. We start from o-SRO for discussion. The o-SRO lattice has the *Pbnm* (*Pnma*) space group and its lattice constants are *a* = 5.57108 Å, *b* = 5.53543 Å, and *c* = 7.8504 Å^14^,^32^. The Ru-O-Ru bond angles (ϕ) are 163.08° (ϕ_Ru-O1-Ru_) and 162.4° (ϕ_Ru-O2-Ru_) with the A-site size *R*_*A*-XII_ ~1.4401 Å where subscript XII refers to the twelve-coordinate value for Sr^2+ ^14,33. It is known that SRO is a popular oxide electrode for its good chemical durability, metallic conductivity, and outstanding epitaxial properties. These properties were demonstrated as early as 1969, including the ferromagnetic magnetic state below ~160 K and metallic transport behavior below 800 K. It seems that these properties in thin film SRO are indeed robust against coherent lattice strain induced by various substrates, showing no big difference from ceramic SRO^34^,^35^,^36^,^37^,^38^. The oxygen deficiency in thin film SRO seems not influential on the electro-transport and magnetism. In this sense, it is usually accepted that SRO is a ferromagnetic metal.

Nevertheless, electro-transport and magnetism of SRO can be either fragile against some other structural and chemical perturbations. One example is the A-site (Sr^2+^ site) and Ru-site chemical substitution by isovalent or heterovalent ions, which has been demonstrated to impose substantial influence on the electro-transport and magnetism. We first consider one extreme end: o-CRO. Different from SRO, the Ca^2+^ ionic radius is *R*_*A*-XII_ ~1.339 Å, making more seriously distorted lattice and more bent Ru-O-Ru bond ϕ ~148° much smaller than that of o-SRO^32^,^39^,^40^,^41^. As a fact of matter, o-CRO would be expected to exhibit antiferromagnetic and insulating behaviors. Indeed, o-CRO loses its ferromagnetism until the lowest *T* but no antiferromagnetic (AFM) order is developed^42^,^43^. Instead, a paramagnetic behavior is identified with a negative Curie-Weiss temperature *Θ*_*W*_ ~ −68 K, suggesting an AFM background. However, o-CRO remains to be a metal although its electrical resistivity (*ρ*) dependence of *T* is much weaker than most metallic transition metal oxides^32^,^44^. In particular, its *ρ* is comparable in magnitude with that of o-SRO, while reported electrical resistivity data from various groups are scattered^32^,^45^,^46^. Here it should be noted that a change of *R*_*A*_ for ~7% regarding o-SRO and o-CRO is not a small quantity, leading to remarkable differences in lattice parameters and distortion between them, as summarized in Table 1 taken from our data and literature^14^,^33^. To our opinion, a comparison between o-SRO and o-CRO is far from sufficient to conclude whether the electronic structure and magnetism of ARuO_3_ are robust or fragile against structural variations.

Similar case can be found if one considers the other extreme end: c-BRO. Given that the Ba^2+^ ionic radius is *R*_*A*-XII_ ~1.6101 Å, an ultrahigh pressure synthesis allowed a meta-stable c-BRO structure with ϕ ~180°, and a ferromagnetism with *T*_*c*_ ~60 K and a metal-like conductivity were identified recently^14^. A distinct character for ARuO_3_ series is that the electrical conductivity shows a dependence of *R*_*A*_ in a way opposite to that of magnetism. Usually, a more distorted perovskite would give rise to a small *W* which favors an antiferromagnetism and probably bad electron itinerancy thus large *ρ*. This hypothesis does not apply to the magnetism of ARuO_3_, noting that it is o-SRO instead of c-BRO to have the highest *T*_*c*_ (strongest ferromagnetism)^32^,^47^. It neither applies to the electrical conduction, noting that it is o-SRO instead of c-BRO to have the smallest *ρ*^28^,^32^,^48^. It is noted again that the differences in lattice parameters between o-SRO and c-BRO are non-negligible. In fact, the causes for ferromagnetism disappearance in o-CRO and reduction in c-BRO with respect to o-SRO, are under hot debate^8^,^14^,^15^,^16^,^30^, which hints some questions on the consensus regarding the evolution of electronic structure and magnetism of ARuO_3_ with intrinsic or external perturbations.

Besides a number of investigations on o-CRO and o-SRO in bulk and thin film forms^16^,^17^,^49^,^50^,^51^,^52^, a series of isovalent substitution experiments on o-SRO at Sr site by Ca^2+^ and Ba^2+^ have been carried out^14^,^17^,^19^,^32^,^33^,^53^,^54^,^55^,^56^,^57^,^58^. The reported results have been somehow authors-dependent but in general the electro-transport and magnetism are sensitive to the substitution of Sr^2+^ by Ca^2+^ or Ba^2+^. We only present a short outline of the consequence of Ba-substitution at low level and Ca-substitution over a broad range. For Sr_1−*x*_Ca_*x*_RuO_3_ (SCRO), the Ca substitution makes the lattice seriously distorted, characterized by reduced ϕ_Ru-O1-Ru_ and ϕ_Ru-O2-Ru_ and stretched Ru-O bond lengths^32^,^33^. While the Ru-Ru AFM exchange as a consequence of the lattice distortion begins to compete with the ferromagnetic (FM) exchange, the unusual fact is that the magnetic susceptibility *χ* at *T* > *T*_*c*_ begins to deviate the Curie-Weiss behaviors in accompanying with the reduced *T*_*c*_^9^,^17^,^32^,^33^,^59^. This reduction is not driven by the enhanced AFM exchange but due to a dilution of the FM exchange, evidenced by the anomalous *χ*(*T*) behavior associated with the appearance of Griffiths (phase) state^14^,^17^,^32^,^33^. The density of states near the Fermi level or in other words the itinerant electron degeneracy does not change much. This scenario seems to explain reasonably the ferromagnetism disappearance and nearly invariable *ρ-x* dependence. For Sr_1−*x*_Ba_*x*_RuO_3_ (SBRO), the Ba substitution gives rise to distinctly different consequence^14^,^58^. In spite of insufficient data, the mystery of Ba substitution has still been gradually unveiled^14^. First, the substitution does not change the Curie-Weiss behavior of *χ*(*T*) above *T*_*c*_. Therefore, one can discuss the physics within the conventional framework of correlated physics. Since Ba^2+^ has bigger ionic size and stronger ionic character than Sr^2+^ has, two physical ingredients are expected^14^. One is the suppressed lattice distortion and then broadening of bandwidth *W*, which would enhance the ferromagnetism and improve the electron itinerancy. The other is the competition between the Ba-O bonding and Ru-O bonding which eventually elongate the Ru-O bond at higher *x* level, making narrow *W*, low *T*_*c*_, and inactive electron itinerancy with increasing *x*. The latter ingredient seems to be dominant for c-BRO, explaining the low *T*_*c*_ and large *ρ* of c-BRO with respect to o-SRO^9^,^32^.

To this stage, one understands that the electro-transport and magnetism of ARuO_3_ do depend largely on a number of structural and electronic factors, such as the A-O bond length, A-site ionic character (e.g. electronegativity), RuO_6_ oxygen octahedral rotation and tilting, and Ru-O bond length etc. Nevertheless, so far every trial by means of isovalent or heterovalent substitution has always caused more than one occurrence of these factors and thus a general scenario for predicting the electro-transport and magnetism of ARuO_3_ and other ruthenates is yet complicated^18^,^19^,^56^,^60^,^61^. In particular, these substitutions inevitably bring into cation disorder and size mismatch which have been demonstrated to play substantial roles in modulating the structural and properties of transition metal oxides. Specifically for ARuO_3_, these disorder-induced phenomena have not yet well understood in spite of some fragmental works available^60^,^61^. For instance, either Ca- or Ba-substitution to o-SRO at low level always brings into random ionic characters/size mismatch which may be called the A-site (cation) disorder. One question associated with this disorder is how the electro-transport and magnetism respond.

The main motivation to address the A-site cation disorder-induced phenomena in ARuO_3_ originates from several aspects. First, the Anderson localization as a well-known concept in condensed matters has been emphasized in correlated electron systems too^62^,^63^. Various approaches to induce disordering in electronic structure may favor the localization effect. Here, the A-site disorder certainly imposes influence on the electro-transport and magnetism. Second, the site disorder in strongly correlated electron systems such as manganites has been extensively demonstrated to play important role in modulating the electro and magnetic properties. The consequence of A-site disorder in ABO_3_ manganites has been well investigated and the itinerant electron localization and spin-glass behavior with increasing disorder were identified^59^,^64^,^65^,^66^,^67^. Similar cation disorder issues can be found in ferroelectric oxides^65^,^68^,^69^ and other magnetic perovskites^70^. Third and for practical applications, o-SRO as a favorable thin film electrode is insensitive to very different deposition conditions and can endure high density of imperfections and deficient defects as a kind of structural disorder^8^,^24^,^60^,^71^,^72^. The underlying physics is also interested.

In this work, we proposed the idea of co-substitution of Ba^2+^ and Ca^2+^ at Sr^2+^-site, keeping the average Sr^2+^-site size invariant but the local disordering is induced, which should increase with increasing substitution level. Different from earlier works on manganites^65^,^68^, it will be shown that o-SRO exhibits unexpectedly high stability against the A-site disorder. This consequence is certainly different from the case of SRO thin films deposited on lattice mismatch substrates where the whole lattice is coherently strained. We will also discuss the underlying physics for such insensitivity of the electrical and magnetic behaviors to the disorder.

## Results

### A-site disorder

For a perovskite ABO_3_, the A-site ionic size disorder is usually measured by the mean square momentum or size disorder variance *σ*^2 ^65,73. For the present case, the A-site contains three types of ions: Sr^2+^, Ca^2+^, and Ba^2+^. The chemical formula becomes Sr_1−*x*_(Ca,Ba)_*x*_RuO_3_ with the co-substitution level *x*. The A-site size variance is:


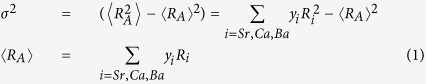


where *y*_*i*_ and *R*_*i*_ are the atomic fraction and ionic radii of *i*-type ions at A-site, and 〈…〉 stands for configuration averaging. In our work, condition 〈*R*_*A*_〉 = *R*_Sr_ at Sr^2+^ state is always maintained in order to exclude effect from variation of the average A-site size. Therefore, we must have the following relationships:


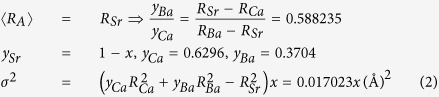


noting that both *σ*^2^ and *x* can be used as a measure of the A-site disorder.

### Microstructural characterizations

**Figure 2:**
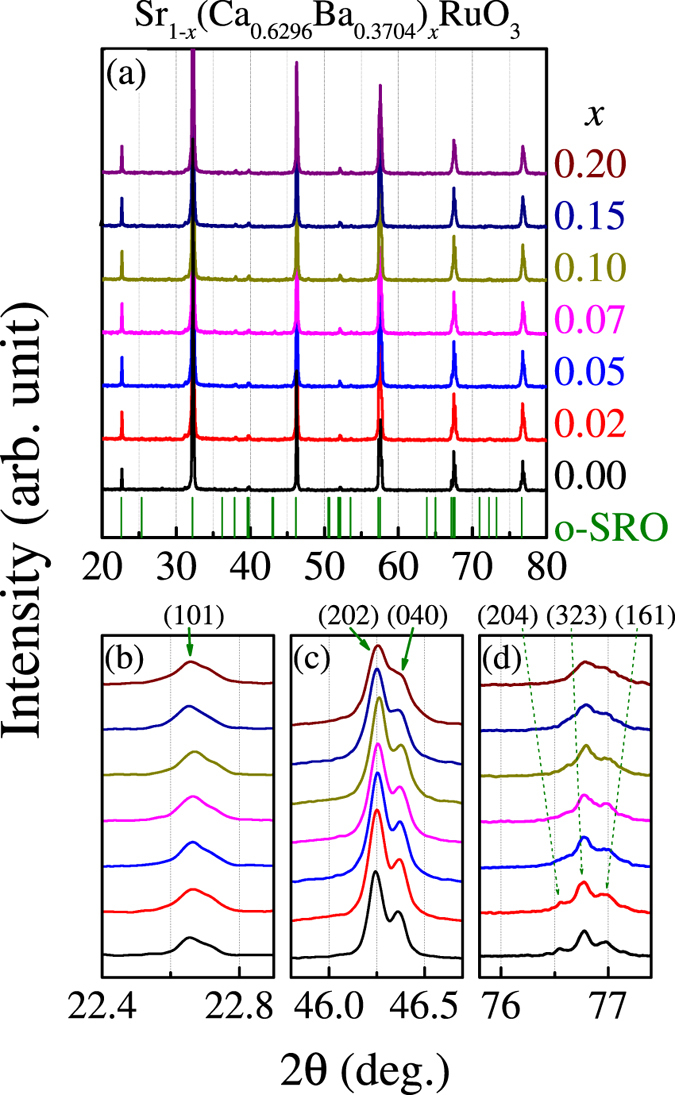
Measured XRD θ–2θ spectra for a series of SCBRO samples with different *x* values marked aside: (**a**) spectra over a wide angle-range, and the local amplified profiles of reflection (101) (**b**), reflections (202) and (040) (**c**), and reflections (204), (323), and (161) (**d**) respectively. The standard o-SRO spectrum is inserted in (**a**) for reference.

Before we investigate the effect of A-site disorder on the electro-transport and magnetism, a characterization of the structural and chemical homogeneity of the as-prepared samples is necessary. First, the X-ray diffraction (XRD) θ–2θ spectra of a set of samples are presented in Fig. 2(a) where the standard XRD reflections from o-SRO are inserted and the values of *x* are marked aside. It is seen that all the peaks for different *x* can be properly indexed by the standard o-SRO reflections without identifiable impurity phase. The relative intensity of each peak shows no obvious dependence on *x*, suggesting the invariable lattice structure with increasing *x*.

For a clearer clarification, several amplified reflections at 2θ ~22.65°, 46.30°, and 76.85° are presented in Fig. 2(b)~(d) respectively. No identifiable shifting of the peaks is observed, indicating that the averaged A-site size does not change with *x*. Second, gradual evolution of the peak profile details with increasing *x* can be detected, taking the reflections (202) and (040) shown in Fig. 2(c) as an example. The well-defined two peaks at *x* = 0 become broadening with *x* and merged at *x* = 0.20, suggesting the local lattice distortion due to the A-site size mismatching. Similar behavior can be seen for reflections (204), (323), and (161), as shown in Fig. 2(d).

**Figure 3:**
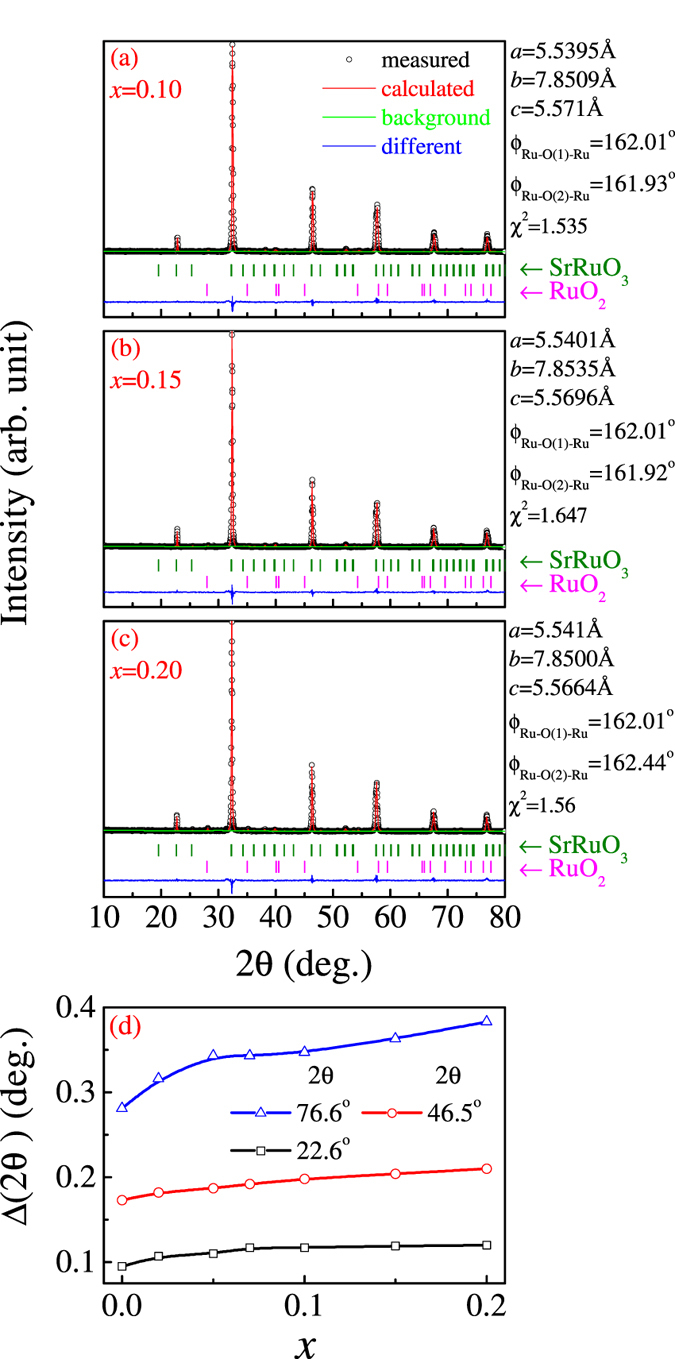
Rietveld refined XRD θ–2θ spectra for SCBRO samples *x* = 0.10 (**a**), *x* = 0.15 (**b**), and *x* = 0.20 (**c**), respectively. The lattice parameters *a*, *b*, *c*, ϕ_Ru-O(1)-Ru_, and ϕ_Ru-O(2)-Ru_, as evaluated from the Rietveld refinement, as well as refinement reliability coefficient χ^2^ are marked aside. The full-width at half maximum Δ(2θ) at 2θ = 76.6°, 46.5°, and 22.6°, as a function of *x*, respectively, are plotted in (**d**).

For a quantitative evaluation of the possible lattice distortion, we adopt the Rietveld refinement package to fit the measured XRD data and the data for three samples *x* = 0.10, 0.15, and 0.20 are presented in Fig. 3(a)~(c) respectively. Here the standard XRD reflections for o-SRO and RuO_2_ are inserted for reference since it was found that sample *x* = 0.20 contains very weak signals from RuO_2_. The best fitted lattice parameters for the three samples are listed aside and the parameters for all the samples are listed in Table 1. It is shown that all the samples have their lattice structure quite similar to that of o-SRO with quite high reliability (*χ*^2^ < 1.8) although fluctuations are inevitable. One may claim that the macroscopic lattice structure of o-SRO remains nearly invariable to the Ca/Ba co-substitutions. Nevertheless, the local lattice delicate distortion with increasing A-site disorder is detectable, characterized by the full-width at half maximum Δ(2θ) data for three 2θ angles as a function of *x*, as shown in Fig. 3(d). As expected, Δ(2θ) increases gradually with *x*, marking the enhanced local lattice distortion from the A-site disorder.

**Figure 4:**
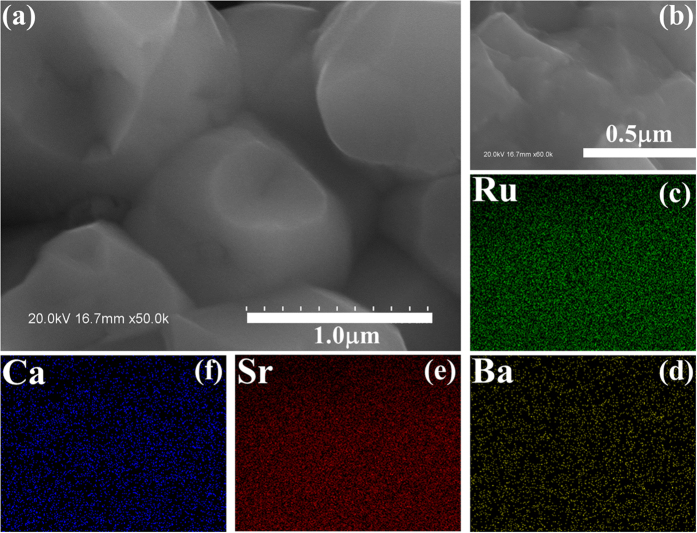
(**a**) The SEM image of the fresh surface for sample *x* = 0.15. The SEM image of a local area is shown in (**b**), and the planar distributions of elements Ru (**b**), Ba (**c**), Sr (**d**), and Ca (**e**) from the electron probe microanalysis are given respectively.

We also checked the cation distribution homogeneity in these samples by electron probe microanalysis to exclude possible chemical segregation. The SEM image of microstructure of sample *x* = 0.15 is presented in Fig. 4(a) at a sub-μm scale, showing dense grain-packing with typical grain size of ~1.0 μm. Subsequently, a small region (Fig. 4(b)) from the SEM imaging window was focused on for composition analysis. The planar distributions of Ru, Ba, Sr, and Ca are plotted in Fig. 4(c–f) respectively. All these cation ions have homogeneous distribution without trace of compositional segregation. The chemical homogeneity remains quite good upon the A-site disorder up to *x* = 0.20.

### Electrical transport and magnetism

**Figure 5:**
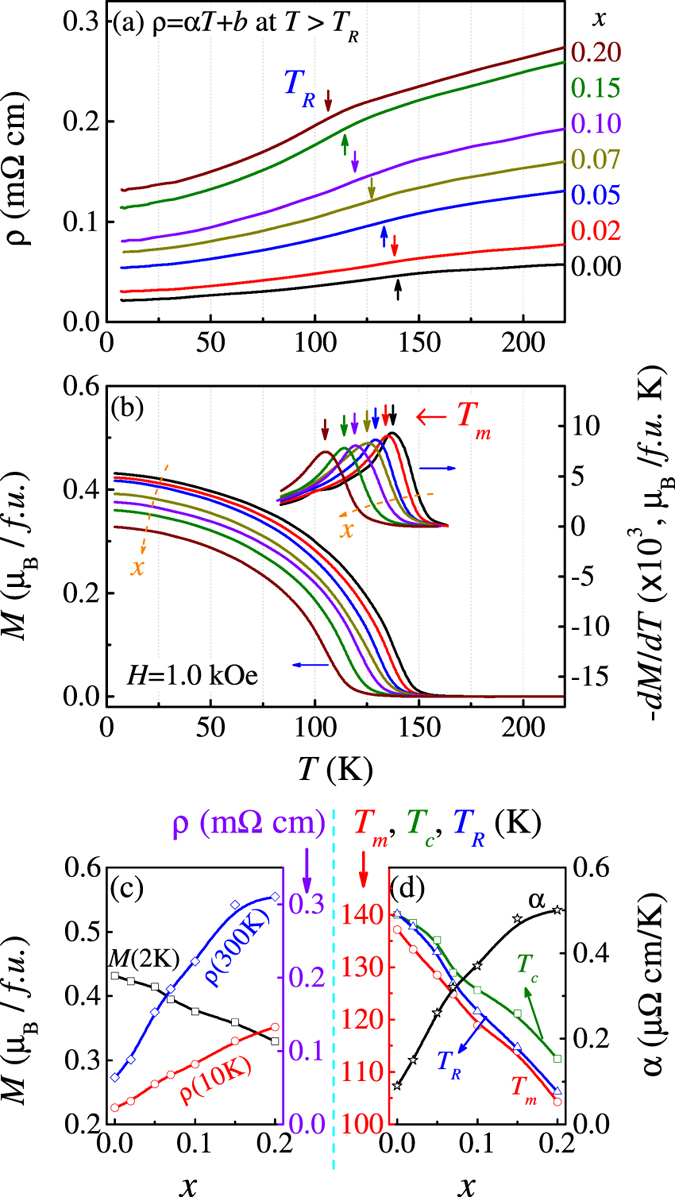
Measured electrical resistivity *ρ*(*T*) and magnetization *M*(*T*) data for a series of SCBRO samples with different *x* are plotted in (**a,b**). Magnetization *M* was measured in the field-cooling mode with cooling-field and measuring field of 1.0 kOe. The evaluated *ρ* at *T* = 10 K and 300 K, evaluated *M* at *T* = 2 K, and parameters *T*_*R*_, *T*_*M*_, *T*_*c*_, *α* etc, as a function of *x*, are plotted in (**c**,**d**) respectively.

Now we turn to the electro-transport and magnetism in response to the increasing A-site disorder. The measured *ρ*(*T*) data for several samples *x* are plotted in Fig. 5(a) where the arrows indicate the anomaly (*T*_*R*_) in the metal-like *ρ*(*T*) curve^14^,^32^. As well known, o-SRO (*x* = 0) exhibits a clear itinerant electron FM transition at *T*_*c*_ ~161 K, responsible for the anomaly at *T*_*R*_^14^,^32^. The corresponding magnetization *M*(*T*) curves are plotted in Fig. 5(b), demonstrating a typical FM transition for all the samples. For each curve, a Curie-Weiss fitting of the paramagnetic data gives rise to the Curie-Weiss point *T*_*c*_, while the *dM*/*dT* data gives the maximal point at *T*_*m*_, as shown in Fig. 5(b) too. These parameters (*T*_*R*_, *T*_*c*_, *T*_*m*_, *α*) together with *M* and *ρ* at several *T* are plotted in Fig. 5(c,d).

**Figure 6:**
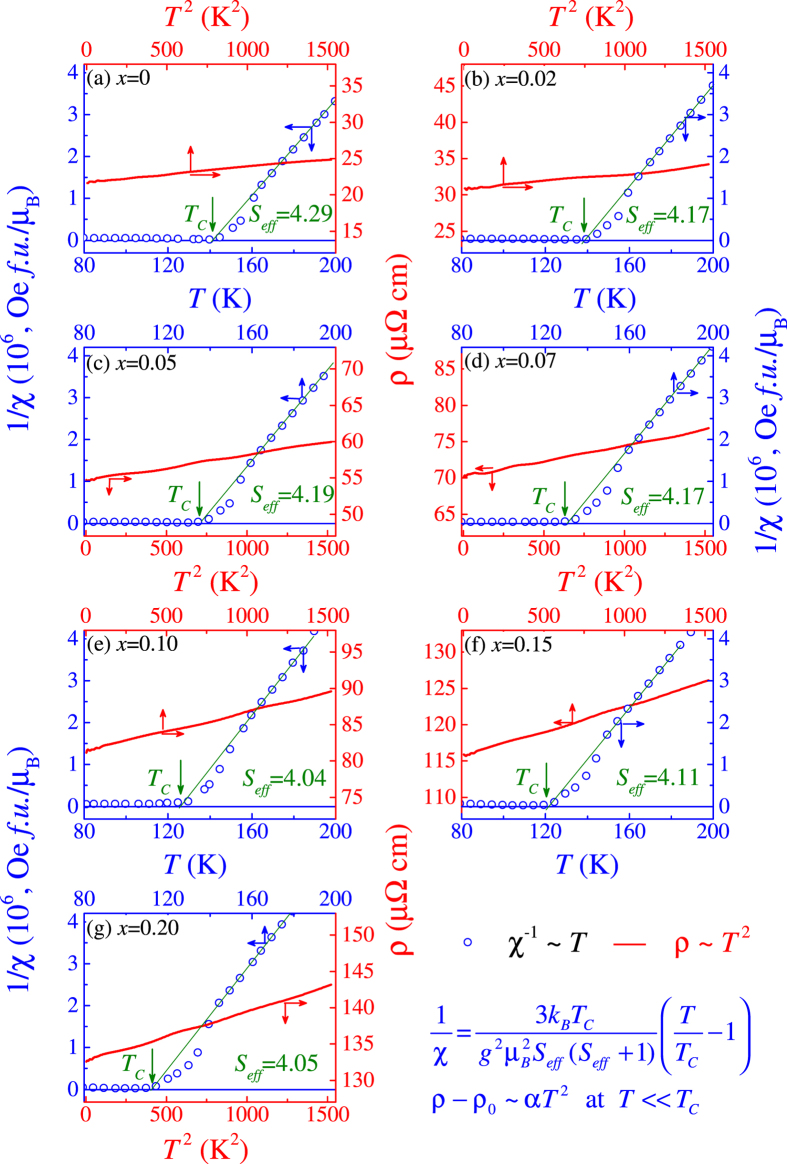
Measured inverse magnetic susceptibility (1/*χ*) (dots) as a function of *T* and electrical resistivity *ρ* (red line) as a function of *T*^ 2^ for various samples. The fine solid lines are the best fitting results. The effective moment (*S*_*eff*_) right above *T*_*c*_ are inserted.

Here several features should be mentioned. First, the increasing A-site disorder shifts the *ρ*(*T*) curve upward and the anomaly leftward simultaneously, leading to slight reduction of *T*_*R*_. However, such a shift is insignificant and the metal-like *ρ*(*T*) dependences remain little changed. Second and similarly, the *M*(*T*) curve also shifts downwards and leftwards with gradual reduction of *T*_*c*_ and *T*_*m*_. Either, the *M*(*T*) dependences remain little change. Third, the linear *ρ* − *T* data above *T*_*R*_ can be fitted by relation *ρ = αT* + *b* with monotonously increasing coefficient *α*(*x*), suggesting more scattering due to the local lattice distortion. Far below *T*_*R*_ (*T* ≪ *T*_*R*_), the *ρ* − *T* data can be reasonably described by relation *ρ* − *ρ*_*0*_ ~ *T* ^2^ (see Fig. 6 below), a symbol of Fermi-liquid system as reported for o-SRO^8^,^14^,^74^. Fourth, the small differences between *T*_*R*_, *T*_*c*_, and *T*_*m*_ and similar dependences of them on *T* indicate that they are from the same physics.

**Figure 7:**
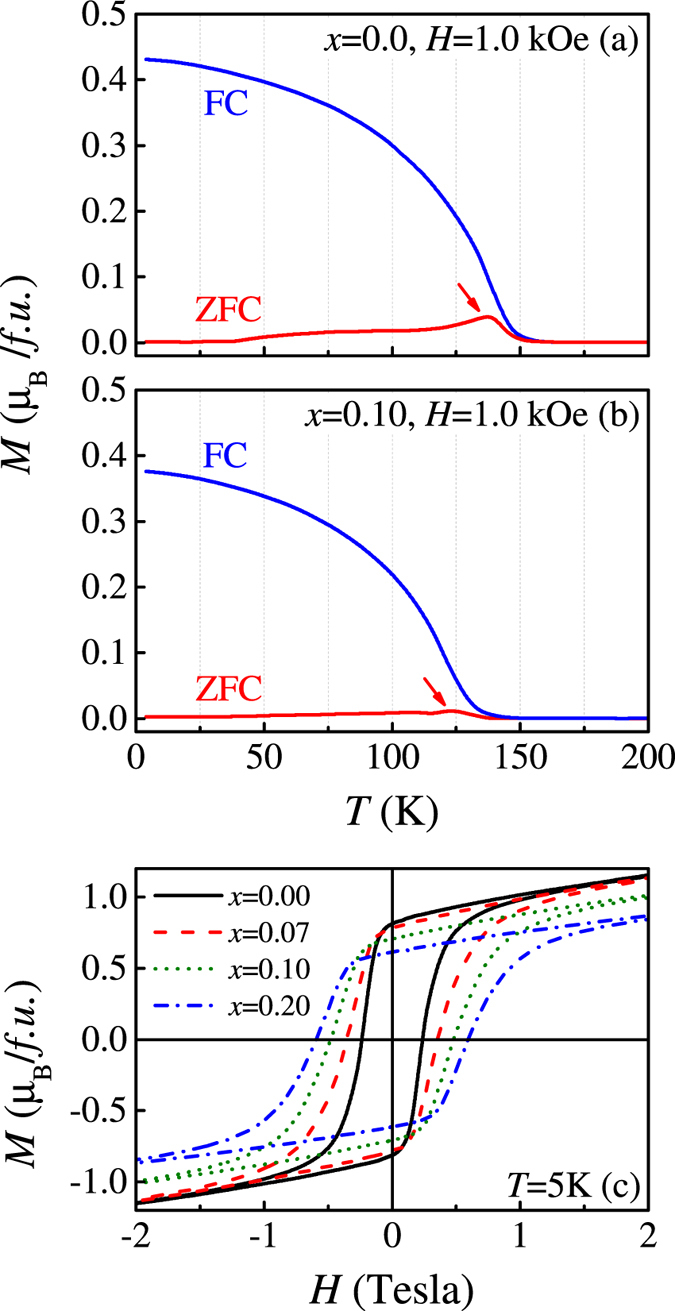
Measured *M*(*T*) data under ZFC and FC modes for samples *x* = 0.0 (**a**) and *x* = 0.10 (**b**) respectively. The measuring field is 1.0 kHz. The measured *M*-*H* loops at *T* = 5 K for various samples are plotted in (**c**).

The effect of the A-site disorder on the ferromagnetism of o-SRO can be illustrated too from the *M*(*H*) dependence. First, the *M*(*T*) curves under the ZFC and FC conditions with cooling and measuring fields of ~1.0 kOe are plotted in Fig. 7(a,b) for *x* = 0 and 0.10 respectively. We see a clear separation of the *M*(*T*) curves under the ZFC and FC modes with a small cusp right below *T*_*c*_ for all the samples including pure SRO. Although SRO is often seen as a ferromagnetic metal, such a feature of ZFC-FC separation in polycrystalline bulk SRO, thin film SRO, and even single crystal SRO, was reported in earlier works^75^,^76^,^77^. Indeed, the FC mode measured *M*(*T*) data do show ferromagnetic transition around *T*_*c*_, but the clear separation in *M*(*T*) curve between the ZFC and FC modes is also a well-established feature. Certainly, such a feature is not necessarily related to spin-glass behavior in the strict sense, one may argue that this ferromagnetism may be sensitive to defects and local lattice distortion inevitable in the samples. Additional investigation is needed, e.g. by measuring the ac magnetic susceptibility and spin relaxation^15^,^32^,^74^,^75^.

On the other hand, the *M-H* loops at *T* = 5K for several samples are plotted in Fig. 7(c). While the saturated magnetization *M*_*s*_ for sample *x* = 0 reaches ~1.2 *μ*_*B*_/*f.u.*, consistent with earlier reports^32^, the A-site disorder suppresses gradually the *M*_*s*_ down to ~0.85 *μ*_*B*_/*f.u* at *x* = 0.20. It is known that Ru ion in ARuO_3_ is in the Ru^4+^ state with electronic configuration *d*^4^. The low-spin (*S* = 1) state contributes an effective moment of ~2.8 *μ*_*B*_ above *T*_*c*_, as predicted by the low-spin moment of 2[*S*(*S* + 1)]^1/2 ^78,79. However, this effect is insignificant and the reduction of *M*_*s*_ is only ~30% given *x* = 0.20, indicating that the magnetism of o-SRO is not as sensitive as expected to the A-site disorder. For the coercive field, it is revealed too that the A-site disorder enhances the coercive field up to 0.65 T at *x* = 0.20. Earlier work revealed that the ferromagnetic SrRuO_3_ thin films deposited on LaAlO_3_ substrates exhibit a coercive field of ~10 kOe, while the coercive field for single crystal SrRuO_3_ is 3 kOe^38^,^80^. There were several reports on polycrystalline SrRu_1−*x*_Cr_*x*_O_3_ (0 ≤ *x* ≤ 0.12) showing substitution-dependent coercive field^81^.

While that the magnetism of SRO is rigidly ferromagnetic or spin-glass like remains to be an issue, one may investigate the stability of magnetism of o-SRO to the A-site disorder by evaluating the spin clustering tendency in the co-substituted samples. We assume the effective moment as *S*_*eff*_, and the *dc* magnetic susceptibility *χ* above *T*_*c*_ can be described by^32^,^65^:


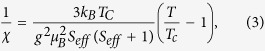


The *χ*^−1^(*T*) data for several samples are presented in Fig. 6 together with the low-*T ρ*(*T*^2^) data. The best fitting of the high-*T χ*^−1^ data using Eq. (3) gives the values of *T*_*c*_ and *S*_*eff*_, as marked in the plots. Several seminal characters can be highlighted. First, for all the samples, the *χ* − *T* data at *T* > *T*_*c*_ can be well described by Eq. (3). And more, the evaluated *S*_*eff*_ is roughly ~4.10 *μ*_*B*_, independent of *x* and bigger than the effective moment of Ru^4+^ (~2.8 *μ*_*B*_)^78^,^79^. This implies that the high-*T* paramagnetic phase favors a spin-cluster state instead of the single-spin ensemble. Second, an obvious deviation of *χ*^−1^ from the fitting as *T* tends close to *T*_*c*_ from the high-*T* side, is identified, suggesting a Griffiths phase-like feature, as extensively discussed for o-SRO and SCRO systems^14^,^17^,^32^, although we have no sufficient evidence with this statement. This feature remains observable for all the samples. Third, the low-*T ρ* data do fit the *T* ^2^-dependence, as shown by the linear solid (red) line for each case^8^,^14^. All these characters allow us to claim that the magnetic properties of o-SRO are robust against the A-site disorder.

### Electrical resistor network model

As a complementary part to the above discussion, one may propose a simplified model to fit the electro-transport behavior quantitatively. Due to the random occupation of the A-site by Sr, Ca, and Ba, the simplest approach is to develop an electro-resistor network and evaluate the *ρ*(*T*) dependence to see its consistence with measured data.

**Figure 8:**
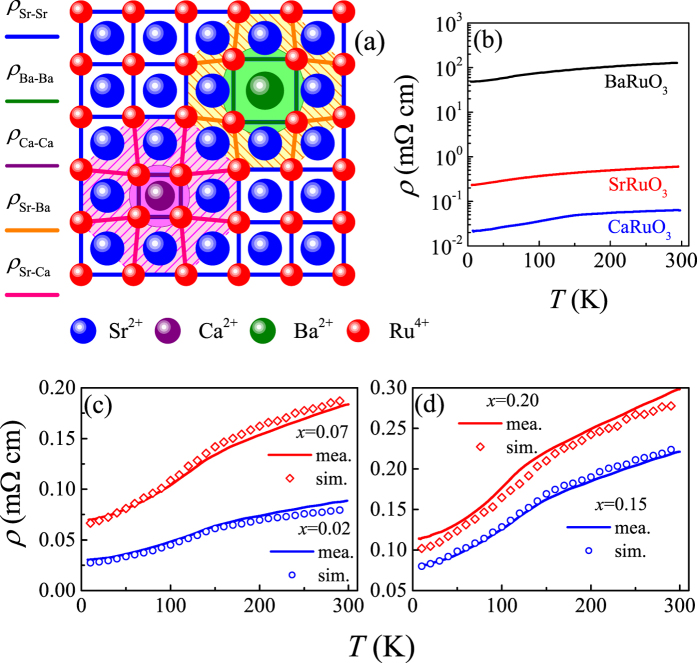
A schematic of the two-dimensional section for a three-dimensional lattice where oxygen ions are omitted for clearance (**a**). The measured *ρ*(*T*) data for polycrystalline SRO and CRO samples are plotted in (**b**) where the *ρ*(*T*) data for polycrystalline c-BRO are taken from ref. 14. The measured *ρ*(*T*) data (mea.) and calculated data (sim.) from the resistor-network model are plotted in (**c**) and (**d**) respectively for samples *x* = 0.07 and *x* = 0.20.

To proceed, one constructs a three-dimensional lattice as shown in Fig. 8(a) for the two-dimensional section. The electro-transport is realized by a resistor network consisting of Ru^4+^-Ru^4+^ bonds in the lattice. Since the highest *x* is 0.20, one may assume that the Ca and Ba occupations in the lattice are well separated without the nearest-neighbored Ba-Ba, Ca-Ca, and Ba-Ca pairing. If one A-site is occupied by ion A′ (=Ca or Ba), it has twelve nearest-neighbor Ru^4+^-Ru^4+^ bonds whose resistivity is assigned as *ρ*_A′-A′_ and twenty-four next nearest-neighbor Ru^4+^-Ru^4+^ bonds whose resistivity are assigned as *ρ*_Sr-A′_. The left are resistors with resistivity *ρ*_Sr-Sr_. It should be noted that any variation of the A-site ionic size would influence the surrounding nearest-neighboring *ρ*_A′-A′_ and the next nearest-neighboring *ρ*_Sr-A′_, and the consideration of *ρ*_Sr-A′_ takes into account partially the itinerary feature of Ru^4+^ electrons. Here, the values of *ρ*_A′-A′_ and *ρ*_Sr-Sr_ at a given *T* are taken from the measured data of polycrystalline o-CRO, c-BRO, and o-SRO respectively. While the data for c-BRO are taken from ref. 14, the *ρ*_Ca-Ca_(*T*) curve is from our earlier work^82^,^83^. It is noted that the electrical resistivity of o-CRO single crystals reported in literature is slightly larger than that of o-SRO^32^,^45^. In the present work, the sintered polycrystalline o-CAO shows slightly smaller resistivity than polycrystalline o-SRO. These *ρ*(*T*) data are plotted in Fig. 8(b) for reference.

Besides *ρ*_A′-A′_ and *ρ*_Sr-Sr_, one has to determine *ρ*_Sr-A′_ (A′ = Ca and Ba) as a function of *T* too. A physically sufficient argument on a derivation of *ρ*_Sr-A′_ is quite challenging since it can be related with the details of electronic structure associated with the lattice distortion which is unavailable to us. Given the fact that o-SRO accommodates the itinerant electron transport, *ρ*_Sr-A′_ can be approximately treated as the geometric mean of *ρ*_Sr-A′_ and *ρ*_A′-A′_, i.e. *ρ*_Sr-A′_ = (*ρ*_Sr-Sr _× *ρ*_A′-A′_)^1/2 ^83,84. In consequence, the *ρ*(*T*) for the three-dimensional cubic resistor networks of different substitution level *x* are evaluated based a sufficient configuration averaging. As examples, the measured (mea.) and evaluated (sim.) data are presented in Fig. 8(c,d) for samples *x* = 0.02, 0.07, and 0.20, respectively. A good consistence of the measured (mea.) and evaluated (sim.) data over the whole *T*-range has been demonstrated, illustrating on the other hand the implication of the resistor network model as a quantitative description of the electro-transport behaviors.

## Discussion

So far, our data from various aspects seem to confirm the high stability of electro-transport behaviors and magnetism against the A-site disorder. The variance up to *x* = 0.20 does not change the qualitative behaviors of o-SRO. Qualitatively, this stability has two-fold origin. On one hand, the Ru^4+^ 4*d*-orbitals are more extended than 3*d*-ones, making the *p-d* hybridization between Ru^4+^ and O^2−^ much less sensitive to the variation of Ru-O-Ru bond lengths and angles^14^,^24^. On the other hand, it was discussed that the stronger ionic character of Ba^2+^ benefits to the bandwidth broadening and thus electrical conductivity. A low-level Ba-substitution (<0.15) benefits to the electro-transport which counteracts to the A-site disorder induced damage to the electrical conductivity^14^,^56^.

Besides, it is known that the A-site size disorder in manganites may drive the electronic phase separation^65^,^85^, which would contribute to remarkable magnetoresistance. For the present SCBRO samples, only negligible magnetoresistance under a magnetic field up to several Tesla was observed. This fact suggest the absence of electronic phase separation which otherwise is effective in enhancing the magnetoresistance.

Finally, as a general remark, it is well-accepted that ARuO_3_ would offer rich quantum states with distinctly different electrical and magnetic properties due to the competitive electron correlation (*U*) and bandwidth (*W*). This argument applies particularly to CaRuO_3_ which is believed to be on the verge of magnetic ordering. Nevertheless, the present work suggests the high stability of o-SRO from the electro-transport and magnetism against the A-site disorder. This high stability can also be a good annotation to the good electrical conductivity of o-SRO as electrode material which is robust against imperfection, defects, and other perturbations^24^,^52^,^60^,^61^.

## Conclusion

In conclusion, we have conducted systematic experiments on the impact of A-site cation disorder (size variance) on the electro-transport and magnetism of polycrystalline o-SRO upon the Ca/Ba co-substitution of Sr. While spatially random Ca/Ba occupation up to *x* = 0.20 has been revealed, it is demonstrated by a series of electro-transport and magnetism characterizations that the itinerant electron ferromagnetism of o-SRO can be maintained, exhibiting the high stability against the sufficient A-site disorder. The possible origin for this stability is discussed, which is the consequence of delicate balance between two local lattice distortion induced effects. One is the Ca-substitution induced AFM background and the other is the Ba-substitution induced bandwidth broadening.

## Methods

We prepared a series of Sr_1−*x*_(Ca_0.6296_Ba_0.3704_)_*x*_RuO_3_ (SCBRO) samples which exhibit different size variance dependent of *x*. It is noted that the Ca/Ba-substitution level *x* is equivalent to the A-site size variance *σ*^2^. For a rough estimation, the size variance corresponding to *x* = 0.20 reaches up to *σ*^2^ ~0.0034 Å^2^, which is on the similar order of magnitude as the size variance of manganites specifically proposed for the A-site size disorder investigations, e.g. *σ*^2^ ~0.005 Å^2^ for Sm_0.55_(Ca_0.6_Ba_0.4_)_0.45_MnO_3_.

The SCBRO polycrystalline samples with *x* up to 0.20 were synthesized using the conventional solid state sintering. The highly purified powders of oxides and carbonates were mixed in stoichiometric ratios, ground, and then fired at 1000 °C for 20 h in air. To reduce the effect of trapped space charges near the grain boundaries of polycrystalline samples, the sample volume density should be close to 100%. The resultant powders were reground and pelletized under 1000 psi pressure to disks of 2 cm in diameter, and then the pellets were sintered at 1300 °C for 24 h in air in prior to natural cooling down to room temperature.

The samples crystallinity and structure were checked by X-ray diffraction (XRD) with Cu *K*_*α*_ radiation at room temperature. The refinement of high resolution XRD data was performed using the standard Rietveld method. The chemical composition and its spatial homogeneity down to nanoscale were checked using electron probe microanalysis installed with scanning electron microscopy (SEM, Hitachi S-4800) which was used to observe the microstructure. The transmission electron microscopy (JEOL 2100F microscope) was also used to characterize the microstructure.

The samples’ magnetization (*M*) as a function of *T* and magnetic field *H* was measured using Quantum Design superconducting quantum interference device magnetometer (SQUID) in zero-field cooled (ZFC) and field-cooling (FC) modes respectively. The cooling field and measuring field were both 1.0 kOe unless stated elsewhere. The electro-transport and specific heat were characterized using physical properties measurement systems (PPMS) from the Cryogenic Co. Ltd and from the Quantum Design Inc. The electrical resistivity *ρ* as a function of *T* and *H* was obtained.

## Additional Information

**How to cite this article**: Wang, Y. L. *et al.* High stability of electro-transport and magnetism against the A-site cation disorder in SrRuO_3_. *Sci. Rep.* **6**, 27840; doi: 10.1038/srep27840 (2016).

## Figures and Tables

**Figure 1:**
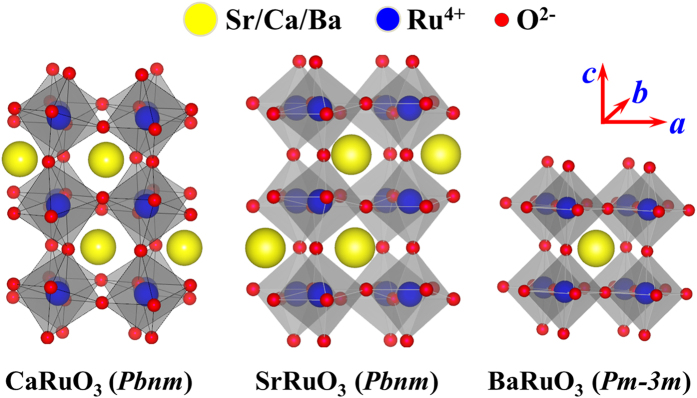
Schematics of lattice structures of o-CRO, o-SRO, and c-BRO, where the space groups of the three compounds are labelled respectively. The lattice distortions are drawn only for a guide of eyes.

**Table 1:** Lattice parameters of o-SRO, o-CRO, c-BRO, and Ca- and Ba- co-doped SRO.

parameters	o-SRO	o-CRO	c-BRO^a^	*x* = 0.05	*x* = 0.10	*x* = 0.15	*x* = 0.20
space group	Pnma	Pnma	Pm-3m	Pnma	Pnma	Pnma	Pnma
*R*_*A*_ (Å)	1.44	1.34	1.61	1.12	1.44	1.44	1.44
*a* (Å)	5.53543	5.53298	4.0059	5.539	5.5395	5.5401	5.541
*b* (Å)	7.8504	7.66333	4.0059	7.8538	7.8509	7.8535	7.85
*c* (Å)	5.57108	5.3574	4.0059	5.5719	5.571	5.5696	5.5664
ϕ_Ru-O(1)-Ru_ (^o^)	163.08	148.6	180	161.995	162.01	162.00	162.02
ϕ_Ru-O(2)-Ru_ (^o^)	162.4	148.7	180	161.924	161.93	191.92	162.44
*χ*^2^	2.3	3.1	2.5	1.769	1.535	1.647	1.56

^a^data from ref. 14.
